# A systematic review of low-cost laparoscopic simulators

**DOI:** 10.1007/s00464-016-4953-3

**Published:** 2016-05-18

**Authors:** Mimi M. Li, Joseph George

**Affiliations:** 1 Faculty of Medicine, Imperial College London, London, UK; 2 Department of Cardiothoracic Surgery, Morriston Hospital, Swansea, UK

**Keywords:** Laparoscopic, Simulation, Trainer, Trainee, Model, Low-cost

## Abstract

**Background:**

Opportunities for surgical skills practice using high-fidelity simulation in the workplace are limited due to cost, time and geographical constraints, and accessibility to junior trainees. An alternative is needed to practise laparoscopic skills at home. Our objective was to undertake a systematic review of low-cost laparoscopic simulators.

**Method:**

A systematic review was undertaken according to PRISMA guidelines. MEDLINE/EMBASE was searched for articles between 1990 and 2014. We included articles describing portable and low-cost laparoscopic simulators that were ready-made or suitable for assembly; articles not in English, with inadequate descriptions of the simulator, and costs >£1500 were excluded. Validation, equipment needed, cost, and ease of assembly were examined.

**Results:**

Seventy-three unique simulators were identified (60 non-commercial, 13 commercial); 55 % (33) of non-commercial trainers were subject to at least one type of validation compared with 92 % (12) of commercial trainers. Commercial simulators had better face validation compared with non-commercial. The cost ranged from £3 to £216 for non-commercial and £60 to £1007 for commercial simulators. Key components of simulator construction were identified as abdominal cavity and wall, port site, light source, visualisation, and camera monitor. Laptop computers were prerequisite where direct vision was not used. Non-commercial models commonly utilised retail off-the-shelf components, which allowed reduction in costs and greater ease of construction.

**Conclusion:**

The models described provide simple and affordable options for self-assembly, although a significant proportion have not been subject to any validation. Portable simulators may be the most equitable solution to allow regular basic skills practice (e.g. suturing, knot-tying) for junior surgical trainees.

The use of laparoscopic surgery has become widely established in clinical practice, with the acquisition of laparoscopic skills now essential for surgical trainees. The technical skills required are, however, distinct from those needed for open surgery; depth perception is impaired due to visualisation on a two-dimensional screen, there is limited tactile feedback, and long laparoscopic instruments create a fulcrum effect and amplify tremor. There is a significant learning curve associated with laparoscopic surgery, and these skills cannot be easily learnt using the traditional apprentice model of surgical training [1].

Simulation is widely regarded as the way forward, and its use has been shown to improve laparoscopic surgical skills in trainees [2, 3]. Simulation offers the opportunity to improve technical skills in a structured, low-pressure environment outside of the operating theatre without risk to patient safety [4]. Different methods of simulation have been described, ranging from high-fidelity virtual reality systems and animal models to low-fidelity box trainers. Box trainers generally have a less realistic interface and are designed for the practice of generic skills required for laparoscopic surgery, such as instrument handling, cutting, and intracorporeal suturing. Virtual reality simulation uses computer-generated graphics and tactile feedback to recreate the operating environment, facilitating practice of procedural-specific skills as well as generic laparoscopic skills [5, 6]. Virtual reality systems are, however, very cost prohibitive and may be inaccessible to many trainees for regular personal use [7]. With the implementation of the European Working Time Directive, opportunities for surgical trainees to gain operative experience in the workplace have also become more limited [8]. A low-cost alternative is needed for trainees to be able to practise and develop their laparoscopic skills outside the workplace. Our objective was to undertake a systematic review of low-cost laparoscopic simulators suitable for home use.

## Methods

A systematic review was undertaken according to PRISMA guidelines [9] to define the properties of low-cost laparoscopic simulators. MEDLINE and EMBASE databases were searched for articles on low-cost laparoscopic simulators published between January 1990 and August 2014. The search terms used were (laparoscopic or thoracoscopic or urological or gynaecological or gynaecological), (simulator or simulation or trainer or training), and (low-cost or home-made or inexpensive or DIY or cheap). Relevant articles from the search were identified by their titles and abstracts; the full paper was then assessed for inclusion. Reference lists for relevant articles were also examined to identify additional studies not identified by the original search.

Articles included were those describing low-cost laparoscopic simulators, which were ready-made or suitable for self-assembly. Articles not written in English, with inadequate descriptions of the simulator, and costs of >£1500 were excluded. The simulators described were categorised into commercial (commercially available or intended for commercial use) and non-commercial (intended for self-assembly). Validation, cost, equipment required, and ease of assembly were examined. For ease of comparison, simulator prices in other currencies were converted into British Pound Sterling using the exchange rate on 16 August 2014. We examined whether any form of validation had been described by the authors. The face validity of each simulator was also rated based on pre-defined criteria for the abdominal cavity and visualisation, giving a score between 0 and 6 (see Table 1).

**Table 1:** Face validity rating system for laparoscopic simulators

**Abdominal cavity**	**Visualisation**
*Enclosed cavity*	* Use of camera*
*Elastic/flexible wall*	*Easily adjustable camera*
*Trocar used at port site*	*Dedicated light source*
A0—does not fulfil any of the criteria	B0—does not fulfil any of the criteria
A1—fulfils 1 criterion	B1—fulfils 1 criterion
A2—fulfils 2 criteria	B2—fulfils 2 criteria
A3—fulfils all 3 criteria	B3—fulfils all 3 criteria
**Total score: **A + B (out of 6)	

## Results

**Fig. 1:**
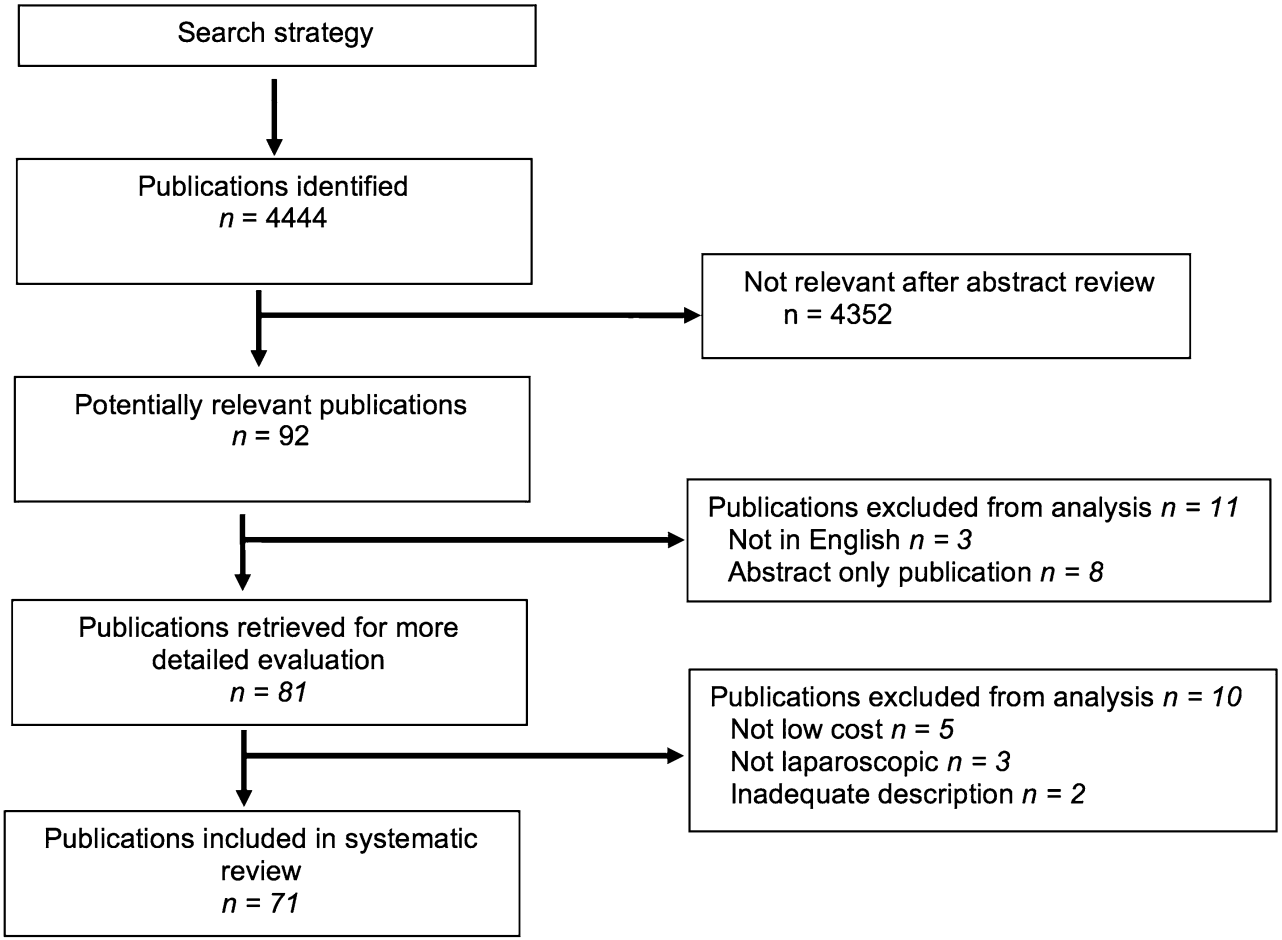
PRISMA flow diagram of study selection for the systematic review

The results of the search are summarised in Fig. 1. 73 unique simulators were identified from 71 articles: 60 were non-commercial (Table 2) and 13 were commercial (Table 3); 55 % (33) of non-commercial trainers were subject to at least one type of validation compared with 92 % (12) of commercial trainers (Table 4). Commercial simulators were already constructed and ready to use, whereas non-commercial simulators required sourcing and self-assembly of materials. The key components required for non-commercial simulator construction were identified as abdominal cavity and wall, laparoscopic port site, light source, visualisation, and camera monitor.

**Table 2:** Non-commercial laparoscopic simulator model comparison: 55 papers describing 57 unique simulators

Paper		Cost	Undergone validation	Face validity Score	Abdominal cavity	Abdominal wall	Port sites	Light source	Visualisation	Camera monitor
1991	Sackier (USA) [32]/	–	Yes	6 (A3 B3)	Custom-made black perspex box; rubber sheet sides	Black perspex	Hole; rubber gasket; trochar	Laparoscope	Laparoscope	Unspecified
1998	Chung (USA) [56]									
1992	Majeed (UK) [33]	–	No	5 (A2 B3)	Metal frame	Black perspex double sheet	Hole; rubber disc; trocar	External lighting	Laparoscope	Video monitor
1992	Mughal (UK) [10]	£75	No	4 (A1 B3)	Opaque plastic storage box	Clear perspex lid	Hole; plastic floor tile; trocar	20 W strip lamps	Laparoscope (or direct vision)	Video monitor
1995	Gue (Australia/NZ) [43]	–	No	3 (A1 B2)	Small coffee table/TV stand	Black plastic sheet; wire mesh	Hole; trocar	Table lamp	Video camera	TV screen
1996	Shapiro (USA) [57]	–	Yes	6 (A3 B3)	Custom-made plastic box	Flexible plastic covering	Hole; trocar	Laparoscope	Laparoscope	Video monitor
2001	Hasson (USA) [58]	–	Yes	6 (A3, B3)	Custom-made metal box	Rubber sheet	Hole; rubber sheet; trocar	Laparoscope	Laparoscope (or camcorder)	Video monitor
2003	Lee (UK) [44]	–	No	4 (A1 B3)	Computer game station (tiered table)	Table top	Anchored trocar	Lamp; external lighting	Camcorder	TV screen
2004	Pokorny (NZ) [11]	NZ $200 (£101.69)	No	4 (A2 B2)	Translucent plastic storage box	Rubber foam sheet over plastic lid	Hole; rubber foam sheet	External lighting	Spy cam; plastic pipe	TV screen
2005	Beatty (UK) [12]	£50	No	2 (A1 B1)	Clear plastic storage box	Clear plastic lid	Hole	External lighting (bright room/lamp)	Webcam	Unspecified
2005	Blacker (UK) [24]	–	No	3 (A1 B2)	Desk drawer	Cardboard	Hole	Desk lamp/strip lamps	Webcam	Desktop computer monitor
		–	No	3 (A1 B2)	Brick-weighted cardboard box	Cardboard	Hole	Desk lamp	Digital camera	Desktop computer monitor
2005	Chung (USA) [25]	–	Yes	2 (A1 B1)	Cut-out cardboard box	Cardboard	Hole	External lighting	Webcam	Laptop
2005 2007	Ricchiuiti (USA) [13]/Bell (USA) [14]	US $360 (£215.70)	No	6 (A3 B3)	Plastic storage box	Plastic lid; plastic sheet	Reinforced hole; neoprene; trocar	Laparoscope/halogen lights	Laparoscope	TV screen
2005	Sharpe (USA) [48]	US $185 (£110.84)	Yes	0 (A0 B0)	Custom-made plastic box	Clear plastic lid	Hole	External lighting	Direct vision	N/A
2006	Chandrasekera (UK) [26]	–	Yes	1 (A1 B0)	Cut-out cardboard box	Cardboard	Hole; trocar	External lighting	Direct vision (unilaterally blinded)	N/A
2006	Do (USA) [59]	–	Yes	5 (A2 B3)	2 large plastic basins	Plastic basin base	Hole; trocar	Lamp	Video camera	Laptop
2006	Griffin (UK) [45]	–	Yes	2 (A0 B2)	Custom-made wooden frame	Thin wooden sheet	Hole	Desk lamp	Camcorder	TV screen
2006 2006	Nataraja (UK) [60]/Nataraja (UK) [61]	–	Yes	3 (A0 B3)	Perspex box	Darkened perspex lid	Hole	Laparoscope	Laparoscope	TV screen
2006	Robinson (USA) [36]	US $50 (£29.96)	Yes	0 (A0 B0)	Custom-made metal box	Metal lid	Hole; unspecified covering material	External lighting	Mirrors	Mirrors
2007	Dhariwal (India) [42]	–	Yes	5 (A2 B3)	Custom-made plastic box	Black plastic lid	Hole; rubber gasket; trocar	Fibre-optic light source	Laparoscope	Video monitor
2007	Haveran (USA) [46]	–	Yes	2 (A0 B2)	Adjustable height posts; wooden sheet	Neoprene; plexiglass frame	Hole	Xenon light source	Camera	TV screen
2007	Martinez (Mexico) [34]	–	No	5 (A2 B3)	Custom-made semi-cylindrical metal box	Metal	Hole; rubber covering	Fluorescent lamp	Video camera; mirror	TV screen
2008	Clevin (Denmark) [62]	–	Yes	5 (A2 B3)	White plastic wash tub	Plastic	Hole; trocar	Laparoscope	Laparoscope	Unspecified
2008	Dennis (UK) [35]	£150	No	4 (A2 B2)	Custom-made wooden box	Plaster of paris	Hole; rubber grommet	Bicycle light	Camcorder	Camcorder screen
2008	Mir (India) [27]	–	No	4 (A1 B3)	Cardboard box	Cardboard	Hole	Laparoscope	Laparoscope	TV screen
2008	Raptis (UK) [15]	£27	No	3 (A2 B1)	Opaque plastic box	Plastic	Hole; trocar	None	Night-vision camera	Computer monitor/TV screen
2008	Sparks (USA) [39]	US $150 (£89.87)	No	3 (A1 B2)	Plywood box; foam board	Plywood hinged lid	Hole	Fluorescent light	Webcam	Laptop
2009	Al-Abed (UK) [16]	£40	No	6 (A3 B3)	Plastic storage box	Foam; latex gloves	Hole; trocar	Halogen light	Webcam; plastic pipe	Laptop
2009	Helmy (Egypt) [40]	–	Yes	4 (A2 B2)	White foam food storage box	Foam box lid	Hole; trocar	Webcam in-built	Webcam	Laptop
2009	Pawar (India) [47]	–	No	3 (A1 B2)	Plywood board box	Plywood	Hole	Tube light	Digital camera	TV screen
2009	Jain (India) [63]	–	Yes	6 (A3 B3)	Custom-made box (unspecified material)	Elastic rubber sheet	Hole; trocar	Laparoscope	Laparoscope	Video monitor
2009	Singh (UK) [28]	–	No	4 (A2 B2)	Shoebox	Cardboard	Hole; trocar	Desk lamp	Digital camera	TV monitor/computer monitor
2010	Jaber (Saudi Arabia) [64]	US $41 (£24.57)	No	2 (A1 B1)	Metallic wire basket; acrylic sheet	Rubber mouse pad	Hole	External lighting	Webcam	Laptop
2010	Rabie (Saudi Arabia) [29]	–	No	3 (A1 B2)	Half large plastic water container; plywood board	Plastic	Hole; trocar	Light bulb	Video camera	TV screen
2010	Rivas (Spain) [17]	–	Yes	4 (A2 B2)	Translucent plastic storage box	Plastic	Reinforced hole; trocar	External lighting	Micro-camera; tube	TV screen
2010	Oliver (UK) [65]	–	Yes	3 (A1 B2)	Cardboard box	Cardboard lid	Hole	Desk light	Webcam	Laptop
2010	Ramalingam (India) [66]	–	Yes	5 (A2 B3)	Custom-made white box (unspecified material)	Box lid	Hole; rubber sheet; trocar/tube	Laparoscope	Laparoscope	TV screen
2011	Alfa-Wali (UK) [30]	–	Yes	3 (A1 B2)	Shoe box	Cardboard	Hole	Torch	Mobile phone camera	Phone screen
2011	Khine (UK) [18]	£60	No	5 (A3 B2)	Translucent plastic storage box	Foldable plastic lid	Hole; neoprene; trocar	Fluorescent light	Webcam	Laptop/desktop computer
2011	Kobayashi (USA) [20]	US $100 (£59.92)	Yes	3 (A2 B1)	Translucent plastic storage box	Plastic lid	Hole; rubber strip	External lighting	Webcam	Laptop
2011	Kiely (Canada) [19] *5 simulators*	C $100-160 (£54.98-£87.97)	Yes	3 (A2 B1)	Translucent plastic storage box	Plastic lid	Hole; trocar	External lighting	Webcam (*various brands*)	Laptop/desktop computer (*various brands*)
2012	Afuwape (Nigeria) [67]	US $34 (£20.37)	No	2 (A1 B1)	Recycled plastic liquid container; plywood board	Plastic	Hole	External lighting	Webcam	Laptop
2012	Bahsoun (UK) [31]	–	Yes	3 (A3 B1)	Cut-out cardboard box; polystyrene	Cardboard	Hole; trocar	External lighting	iPad camera	iPad screen
2013	Akdemir (Turkey) [68]	–	Yes	4 (A1 B3)	Custom-made plastic box	Plastic	Hole; trocar	Laparoscope	Laparoscope	Video monitor
2013	Hennessey (Australia) [69]	–	No	2 (A1 B1)	None	Laptop lid	Trocar; string; skirt hanger	External lighting	Webcam	Laptop
2013	Moreira-Pinto (Portugal) [21]	€33.67 (£26.99)	Yes	4 (A3 B1)	Translucent plastic storage box	Cut-out plastic lid; rubber sheet	Hole; trocar	External lighting	Webcam	Laptop
2013	Omokanye (Nigeria) [41]	–	No	4 (A2 B2)	Plywood box	Box lid	Hole; foam piece	Camera in-built; light bulb	IR CCTV Camera	TV screen
2013	Ruparel (USA) [37]	US $5 (£3.00)	Yes	1 (A0 B1)	Ring binder	Ring binder	Hole	External lighting	iPad camera	iPad screen
		US $5 (£3.00)	Yes	2 (A1 B1)	Cut-out cardboard box	Cardboard	Hole	External lighting	iPad camera	iPad screen
2013	Smith (UK) [70]	US $100 (£59.92)	No	4 (A2 B2)	Plastic crate, plywood and cork sheet	Plastic	Hole; trocar; plastic rings	LED lamp	Webcam	Laptop
		US $130 (£77.89)	No	5 (A3 B2)	Upgraded version: add plywood frame and foam pads to port site					
2013	Wong (USA) [71]	US $309 (£185.14)	Yes	4 (A2 B2)	Custom-made hard plastic box	Vinyl membrane glued to plastic frame	Hole; trocar	LED strip	Miniature CCD camera	Video monitor
2014	Beard (USA) [22]	US $85 (£50.93)	Yes	3 (A2 B1)	Translucent plastic storage box	Plastic lid	Hole; flexible material cover	External lighting	Webcam	Laptop
2014	Escamirosa (Mexico) [38]	–	No	2 (A1 B1)	Clear plastic document case	Plastic	Hole	External lighting	Smartphone or tablet camera	Video monitor
2014	Walczak (Poland) [23]	US $51 (£30.56)	No	3 (A2 B1)	Translucent plastic storage box	Opaque plastic lid	Hole; rubber sheet; metal washer; trocar	LED light bulb	Mirrors	Mirrors
		US $99 (£59.32)	No	5 (A3 B2)	Translucent plastic storage box	Opaque plastic lid	Hole; rubber sheet; metal washer; trocar	LED light bulb	Webcam	Home computer

**Table 3:** Commercial laparoscopic simulator model comparison: 16 papers describing 14 unique simulators

Paper		Simulator	Price	Validation	Face validity
1998	Derossis [72]/Keyser [73]	USSC Laptrainer	–	Yes	6 (A3 B3)
2000					
2000	Scott [74] /Nakamura [55]	Karl-Storz	–	Yes	6 (A3 B3)
2011					
2003	Adrales [75]/Adrales [76]	US Surgical Trainer	–	Yes	5 (A2 B3)
2004					
2005	Waseda [77]	Tuebinger MIC Trainer (Richard Wolf GmbH)	–	No	6 (A3 B3)
2007	Hruby [49]	EZ Trainer	$600 (£359.50)	Yes	1 (A0 B1)
2008	Dayan [78]/Boon [79]	Simulab Laptrainer	–	Yes	3 (A0 B3)
2008					
2008	Singh [80]	iSim	–	Yes	3 (A1 B2)
2010	Hull [81]	Body Torso Trainer BTS300D (Pharmabotics)	£390 ($585) + £975 for Box trainer	No	6 (A3 B3)
2011	Nakamura [55]	Ethicon TASKit	–	Yes	6 (A3 B3)
2013	Xiao [51]/Xiao [52]	Ergo-Lap	$500 (£299.58)	Yes	5 (A2 B3)
2014					
2014	Yoon [53]	iTrainer	$100 (£59.92)	Yes	1 (A0 B1)
2013	Hennessey [50]	eoSim	$750 (£449.37)	Yes	3 (A1 B2)
		FLS simulator	$1680 (£1006.58)	Yes	5 (A3 B2)

**Table 4:** Comparison between commercial and non-commercial simulators

	Non-commercial simulators	Commercial simulators
Unique simulators	60	13
Price range	£3.00–£215.70	£59.92–£1006.58
Subject to validation (%)	33 (55 %)	12 (92 %)
Average Face Validity Score	3 (A2 B2)	5 (A3 B2)

### Abdominal cavity and wall

Materials used to simulate the abdominal cavity aimed to prevent direct vision of the laparoscopic instruments; 68 % (41) of non-commercial simulators utilised off-the-shelf components for the abdomen, whilst 32 % (19) required a custom-made box. The commonest off-the-shelf component was a plastic storage box for the abdominal cavity, with the box lid serving as the abdominal wall [10–23]. Cardboard boxes were also commonly utilised [24–31].

### Laparoscopic port site

The majority of non-commercial simulators (97 %, 58) required creating a hole in the abdominal wall material (by cutting, drilling or piercing) for the laparoscopic port site. Instruments could then be inserted directly into the cavity or through a trocar. Use of a flexible covering material, such as neoprene [13, 18], and ring reinforcement around the port site [13, 32–35] were also described as methods to increase simulator authenticity.

### Primary light source

An adequate light source was required to visualise the interior of the abdominal cavity. External lighting was used for 38 % (23) of non-commercial simulators, particularly where boxes were made from a translucent material [11, 12, 17, 21] or had open sides [36–38]. This was useful in cost reduction, as no additional equipment was required to provide lighting in these cases. The built-in light source from the laparoscope itself provided lighting for 17 % (10) of simulators, desk lamps for 13 % (8), and light-emitting diodes (LED) for 8 % (5). Other lighting methods described included fluorescent lights [18, 34, 39], webcam in-built [40, 41], fibre optics [42], and torchlight [30].

### Visualisation and camera monitor

Visualisation for non-commercial simulators was most commonly achieved using a webcam (37 %, 22) or laparoscope (22 %, 13). Other cameras types described included video cameras [29, 34, 43–45], digital cameras [24, 28, 46, 47], and tablet/smartphone cameras [30, 31, 37, 38]. Direct vision (full [10, 48] or unilaterally blinded [26]) and mirrors [23, 36] were non-electronic methods of visualisation described. Where electronic visualisation was used, a laptop computer, video monitor, tablet, or smartphone were prerequisite and not included in any cost estimates; this was true of both commercial and non-commercial simulators; 40 % (24) of models described use of a laptop/desktop computer screen and 38 % (23) described using a television or video monitor.

### Cost

Forty-six percentage (26) of non-commercial and 54 % (6) of commercial simulators provided a figure for cost. For non-commercial, this was the cost of materials and assembly (e.g. custom-made parts); for commercial simulators, the cost represented the current or intended retail price. The cost ranged from £3 to £216 for non-commercial simulators and £60 to £1007 for commercial simulators. The cost of laparoscopic equipment (instruments and laparoscope) was not included in cost estimates for non-commercial simulators. However, a number of articles suggested that used or expired disposable instruments could be obtained from the operating department at no cost to the trainee [16, 23–26, 39, 40, 44]. Alternatively, they could also be obtained by donation from laparoscopic equipment manufacturers [15, 20, 26]. Electronic devices for visualisation (video monitor, laptop computer, tablet/smartphone) were not included in cost estimates for non-commercial simulators. Laparoscopic equipment and visualisation monitors were also not consistently included for commercial simulator model packages [49–52].

### Face validity

Commercial simulators had better face validity than non-commercial simulators, with a median score of 5 compared to 3 (maximum 6). Commercial simulators tended to utilise higher-fidelity visualisation equipment, with a median visualisation score of B3 compared with B2 for non-commercial simulators. For the abdominal cavity, there was comparable face validity, with both groups having a median score of A2.

## Discussion

Cost will undeniably be a key factor in the accessibility of a simulator model. Many articles omitted cost estimates, so there is difficulty in making a true cost comparison between commercial and non-commercial simulators available. Although there is an overlap in the price range, non-commercial models appear to be able to achieve a lower cost than commercial ones, with the lowest reported figure being $5 (£3) compared to $100 (£60) for a commercial model [37, 53]. This difference could be due to commercial models factoring in a profit margin and assembly fee in addition to the value of the raw materials. Moreover, commercial models will usually include expensive laparoscopic instruments in the cost, which could potentially be obtained cost-free when self-assembling [16, 23–26, 44].

Non-commercial models commonly utilised off-the-shelf components—a potentially a cost-reductive strategy, as custom-made parts could incur a greater expense. In particular, the use of a translucent plastic box provided a sturdy frame and utilised external lighting, negating the need for an additional light source inside the box [11, 12, 17, 21]. Visualisation using a webcam and computer offered an inexpensive solution, as they can be obtained cheaply. With computer ownership being widespread [54], it can be assumed that most trainees have access to a computer at home. Many trainees may also own a tablet computer. Tablet-based simulation could provide a video feed more comparable in quality to a laparoscope than a budget webcam [31]. Using a tablet or smartphone, where the screen and camera are on the same device, may also be easier to assemble. However, adjustment of camera position would be more difficult.

Commercial simulators, although seemingly costlier in comparison, do have the advantage that they come assembled and ready to use, with more models having undergone some form of validation. However, the appropriateness of the validation methods undertaken are not easily assessed, and only models from established industry suppliers appear to have undergone more extensive validation [50, 55]. In terms of face validity, commercial simulators largely seem to have better face validity, particularly as laparoscopes are more frequently used for visualisation, allowing realistic image quality and camera motion. A laparoscope may be difficult to obtain at a reasonable cost; an alternative may be to use a small camera mounted on a plastic pipe, which also allows adjustment of the operative field view [11, 16, 17]. The ideal simulator would have a highly realistic user interface and allow development of both the technical and non-technical skills required for laparoscopic surgery. The simulators examined in this review chiefly aim to develop basic laparoscopic skills such as instrument handling and cutting; therefore, a highly realistic user interface, as in virtual reality simulators, may be superfluous to requirements. However, use of lower-fidelity simulators does not preclude the development of non-technical skills. For example, the simulator could be incorporated into an operating theatre environment with other team members present, where trainees could be observed and assessed on emergency or elective scenarios.

Of course, simply having access to a simulator does not equate to improvement in surgical skill. Regular use of the trainer with feedback from a supervisor would be ideal. Simulator training could take place during the normal working day with allocated practice time, or this could be done at leisure at home.

## Conclusion

The models described provide simple and affordable options for self-assembly, although a significant proportion has not been subject to any validation. Whilst simulation cannot replace operating theatre experience, portable simulators may be the most equitable solution to allow regular basic skills practice (e.g. intra-corporeal suturing, knot-tying) for junior surgical trainees.
